# Disentangling factors that shape the gut microbiota in German Shepherd dogs

**DOI:** 10.1371/journal.pone.0193507

**Published:** 2018-03-23

**Authors:** Åsa Vilson, Ziad Ramadan, Qinghong Li, Åke Hedhammar, Arleigh Reynolds, Julie Spears, Jeff Labuda, Robyn Pelker, Bengt Björkstén, Johan Dicksved, Helene Hansson-Hamlin

**Affiliations:** 1 Department of Clinical Sciences, Swedish University of Agricultural Sciences, SE Uppsala, Sweden; 2 Nestlé Purina Research, One Checkerboard Square, Saint Louis, MO, United States of America; 3 the Institute of Environmental Medicine, Karolinska Institutet, Stockholm, Sweden; 4 Department of Animal Nutrition and Management, Swedish University of Agricultural Sciences, SE Uppsala, Sweden; Wageningen Universiteit, NETHERLANDS

## Abstract

The aim of this study was to explore the development of the gut microbiota in 168 German Shepherd dogs (30 litters) from 7 weeks to 18 months of age and furthermore, to study the effect of relatedness, maternal microbiota composition and living environment in a large and well-defined population of dogs. Using 454 pyrosequencing, we assessed the effects of pre- and postnatal probiotic supplementation (*Lactobacillus johnsonii NCC533 (La1))* and analysed whether administration of the probiotic strain influenced fecal microbiota composition in a placebo controlled double-blinded study. The bitches were treated with probiotics or placebo during last trimester of pregnancy and until their puppies were 8 weeks old, the puppies received the same treatment as their mothers between 3–12 weeks of age. Samples from bitches were collected at pregnancy day 42, partum, 4 weeks postpartum and 7 weeks postpartum and from puppies at the age 4 weeks, 7 weeks, 12–13 months and 15–18 months. Serum IgA, total serum IgE, fecal IgA and IgG antibody responses against canine distemper virus were analysed by ELISA in order to detect any immune stimulating effects of the probiotic strain. Analysis of the fecal microbiota composition showed that the predominant phyla were the same in 7 weeks old puppies as in pregnant and lactating bitches (Firmicutes, Fusobacteria, Bacteroidetes). Proportions among different bacteria as well as diversity varied from 7 weeks old puppies up to 15–18 months of age. Litter mates had a more similar fecal microbiota compared to unrelated dogs and 7 weeks old puppies were more similar to their mothers than to unrelated bitches at 7 weeks postpartum but not at partum. We observed a change in the relative abundance of different bacteria during lactation, and an increase in diversity from pregnancy to end of lactation. The microbial diversity was affected by living area where dogs living in big cities had higher diversity compared to dogs living at the countryside. However, we were not able to demonstrate an effect by pre and postnatal exposure to *Lactobacillus johnsonii NCC533 (La1)* upon the diversity or composition of the microbiota or the levels of serum IgA, total serum IgE, fecal IgA or vaccine response. Our findings provide a better understanding of the canine fecal microbiota in growing dogs as well as in pregnant and lactating bitches. This information forms a basis for further research on the connection between early gut colonization and immune function later in life.

## Introduction

The distal gut microbiota in mammals is characterized by high population density with a great amount of diversity. It forms a closely integrated ecosystem which plays a vital role in the function of the immune system in dogs as well as in other mammals. Knowledge of the canine gut microbiota has improved with the increased throughput and reduced cost of next-generation sequencing technologies [1]. Despite this, not much is known about what impact different environmental factors have on the canine microbiota. To date, the analyses have been limited to cross-sectional analyses and represent few breeds [2–4]. Hand et al. [4] compared the fecal microbiota among closely related dogs using 454-pyrosequencing and showed that genetically related dogs have a more similar fecal microbial composition compared with unrelated dogs of the same breed. It has also been shown that the gut microbiota is influenced by age [2], but it is not clear how it changes from puppyhood to adult age. Nor is it known how the gut microbiota correlates between pregnant and lactating bitches, and their puppies.

The prevalence of allergy and other immune-related disorders have increased in dogs and humans, particularly during the past decades, which is proposed to be a result of a decreased microbial exposure that provides a strong environmental signal for postnatal maturation of the immune system [5,6]. Exposure early in life to immune regulatory bacteria colonizing the gastrointestinal tract is proposed to have life-long consequences in humans [7,8]. Björkstén [6] suggested that microbial diversity is an essential environmental signal for maturation of the human immune system.

It is essential to our understanding of immune-related disorders in both dogs and humans that we study important factors in shaping the microbiota and maturation of the immune system. In this respect, probiotic bacteria are of particular interest. Probiotics are defined as live microorganisms with documented beneficial effects on health [9]. They are of interest as potential modulators of immunity and in prevention and treatment of immune mediated disorders, such as allergies.

Probiotic supplementation in dogs early in life could provide an important tool for modulation of immune function well into adulthood [10]. The immune modulatory effects of early probiotic supplementation in a larger population of free-living dogs have not yet been described. Since the German Shepherd dog is predisposed to immune-related disorders [11], this breed is a logical choice as a model to study the maturation of different immune parameters, the fecal microbiota and the effect of probiotic supplementation.

The aim of this study was to explore the development of the gut microbiota in German Shepherd dogs from 7 weeks to 18 months of age and furthermore, to study the effect of relatedness, maternal microbiota composition and living environment in a large and well-defined population of dogs. Additionally, we evaluated whether administration of probiotics (*Lactobacillus johnsonii NCC533 (*La1*)*) would enhance IgG antibody responses against canine distemper virus (CDV) in serum, as well as the levels of serum IgA, total serum IgE and fecal IgA. Furthermore we assessed the effects on the fecal microbiota of early age probiotic supplementation to bitches and puppies.

## Material and methods

### Animals and treatment

Thirty pregnant German Shepherd bitches from the kennel of the Swedish Armed Forces (SAF) were recruited at 42 days of pregnancy and the bitches as well as their offspring alive at seven weeks of age (n = 184 puppies) were included in the study (Table 1). Consent was received from the kennel facility manager to use the bitches and their puppies in the study. The bitches lived with private families and arrived at the kennel at pregnancy day 37 or earlier. At the kennel, each bitch and her litter had a separate room (9m^2^) in room temperature and with access to outdoor kennel (9m^2^) without any direct contact to other dogs. The bitches were walked in leash three times daily and the puppies were socialized daily by the staff at the kennel. They had free access to water and were fed three-four times daily. All dogs at the kennel were examined weekly by a veterinarian. Upon arrival they were gradually introduced to the diet (Nestlé Purina Pro Plan Puppy Sensitive Skin, Salmon & Rice Dry (32% protein, 20% fat, 1.2% omega 3) used throughout the study. Twenty of the bitches were imported, mainly from other European countries except one from the US, while nine were born at the kennel and one at another Swedish kennel. Twenty-one sires were used for the 30 litters. Four of the sires were imported, seven were from other Swedish kennels, and the rest were born at the SAF kennel. All bitches and their litters were housed and reared with identical routines at the kennel. When the puppies were eight weeks old, they were moved from the kennel to live with families throughout Sweden where living areas were registered as countryside (an area localized between cities) (n = 73), small city (population <200,000) (n = 85) or big city (population >200,000) (n = 26).

**Table 1:** Information of the study population with number of dogs in each treatment group (probiotic (La1) and placebo).

	La1	Placebo	Total
**No. of litters**	15	15	30
**No. alive at birth**	106	87	193
**Stillborn**	10	18	28
**No. at 7w**	101	83	184
**No. caesarean sections**	1	1	2
**Sex distribution (F/M)**	59/42	39/44	98/86
**Birth weight (g)**	499	519	507

Mothers and puppies were restricted to the same diet during the entire study period. Mothers and their litters were separated from other dogs at the kennel and were not given access to other food. All dogs followed the same vaccination program. The bitches were routinely vaccinated every third year against parvo and CDV before time of enrolment, with the last vaccination within three years. Puppies were vaccinated with the live vaccine Nobivac DHPPi vet. at 7 weeks of age, at 12 weeks and at 12–13 months of age (all after sample collection).

### Intervention by probiotic supplementation

The pregnant bitches were divided into two equally sized groups (n = 15) through block randomization (block size 6), where one group received probiotic supplementation (*Lactobacillus johnsonii*, La1) and the other group placebo (maltodextrin). The bitches started on treatment three weeks prior to estimated parturition (pregnancy day 42), and continued until the puppies where eight weeks old. Puppies received oral treatment (same as their mother) at the age of 3 weeks at the onset of exposure to solid food. The treatment continued until the puppies were 12 weeks old. The dogs were treated orally once daily with 0.55g (10^10^ CFU) powder (or poured on the food after 8 weeks of age). The number of active *L*. *johnsonii* was 1.9*10^10^CFU/g.

The chosen probiotic strain was first identified as *L*. *acidophilus* but was reclassified to *L*. *johnsonii* in 1995. It was isolated from the fecal microbiota of a healthy male human. Studies have shown that the bacteria survives the gastrointestinal tract and enhance immune function in mice [12,13].

The study was approved by the Local Animal Ethical Committee in Uppsala, Sweden (C355/9).

### Time for sample collection

Blood and feces were collected from bitches at pregnancy day 42, 12-24h after completed whelping (referred to as partum), 4 and 7 weeks postpartum. Blood and feces were collected from puppies at 7 weeks, 12–13 months (median 12 months and 20 days) and 15–18 months (median 16 months and 16 days) of age.

Serum IgA, IgE and IgG against CDV were measured in puppies at the age of 7 weeks, 12–13 months and 15–18 months, and bitches at 7 weeks postpartum.

### Assessment of fecal microbiota

The fecal microbiota was assessed by 454-pyrosequencing of 16S rRNA genes in puppies at 7 weeks, 12–13 months and 15–18 months of age and from bitches at pregnancy day 42, partum and 7 weeks postpartum. The fecal samples were collected by rectal swabs and frozen at -80°C within 48 hours. Between collection and freezing at -80°C, the samples were stored on dry ice or in -25°C freezer.

#### DNA extraction and preparation of libraries for 454-pyrosequencing

Fecal swabs were placed in a 15 ml conical tubes containing 1.5 ml PBS (0.85% NaCl, 120 mM NaH_2_PO_4_, pH = 8.0) and allowed to set for 5–10 min to loosen. The samples were then vortexed 2–3 times to remove as much of the fecal materials as possible. Samples were transferred to 2 ml tubes and the 15 ml tubes were rinsed with an additional 0.3 ml PBS, which was added to the original sample. After centrifugation at 13,000 x g for 2 minutes. The supernatant was discarded and the pellet was resuspended in 450 μL of Solution CB1 (warmed to 55°C) by vortexing at max speed for 5 minutes using a MoBio Vortex Adapter. Lysates were transferred to the 2 ml Microbead Tubes and the DNA was extracted following the BiOstic Bacteremia DNA Isolation Kit (MoBIO Laboratories, Inc. Carlsbad, CA) protocol. Final Volume of the Eluate was approximately 100 μL. 1 μL of the Genomic DNA was quantified using the Quant-It PicoGreen dsDNA Assay kit (Invitrogen) on an Fl_x_ 800 Microplate Fluorescence reader (Bio-Tek Instruments, Inc.) using Gen 5 software v 2.00.18. Additionally a 1:10 dilution was run on a 1% E-gel (E-gel 96 1% Agarose (GP), Invitrogen cat), with a Ladder (Invitrogen E-gel 96 High range Marker) for 12 minutes (EG setting). Bands were imaged for 700–900 ms on a SynGene G:Box with a Transilluminator using GeneSnap 7.12.06 Software to detect any degradation of the gDNA. Amplicons from 16S rRNA gene was sequenced using 454 at the Core for Applied Genomics and Ecology, University of Nebraska, Lincoln. The V123 and V456 region of the 16S rRNA gene was amplified using bar-coded fusion primers[14] with the Roche-454 A or B titanium sequencing adapters. All samples were multiplexed with equal amount and sequenced using Roche GS FLX pyrosequencer at the Core for Applied Genomics and Ecology, University of Nebraska, Lincoln.

#### Bioinformatics data processing

The raw data from 454-pyrosequencing were processed using QIIME version 1.8.0 [15]. Data were filtered to remove low-quality reads not meeting the following quality criteria: (1) a complete barcode sequence immediately followed by a forward primer sequence, with no mismatch in either barcode or primer sequence; (2) read lengths between 200 and 500 base pairs (bps); (3) average quality score of 25 or higher in a sliding window of 50 bases; and (4) maximum homopolymer run of 6. Sequencing errors characteristic to pyrosequencing were removed by flowgram clustering [16]. Chimeric sequences generated due to PCR amplification of multiple sequences were removed using UCHIME [17]. Processed reads were then demultiplexed into barcode-indexed samples. The barcode, forward primer, and reverse primer were subsequently trimmed from each read. This yielded a total of 1502990 reads from 507 samples from puppies and 246464 reads from 90 samples from bitches. The average length of the reads was 427 and 338 bps for bitches and puppies respectively.

Reads were clustered into operational taxonomic units (OTU) using a closed reference-based UCLUST algorithm at a 97% sequence similarity level implemented in QIIME [15,17]. The reference sequences and taxonomy assignment map were constructed from the greengenes database, August 2013 release [www.greengenes.lbl.gov].

### Sampling and measurement of antibodies in fecal contents

Fecal IgA was extracted from fresh (mothers) or frozen (puppies) feces (due to practical reasons). Fecal samples from puppies were frozen within two hours of collection and extracted in association with analysis, while the fresh fecal samples from the mothers were extracted within 45 min and then frozen. Using 1.5ml of the extraction buffer (50mM-EDTA and 100μg/l soybean trypsin inhibitor in PBS/1% BSA from Sigma-Aldrich, Schnelldorf Germany), 0.5g of faeces were vortexed. Phenylmethanesulphonyl fluoride (25μl, 350mg/l from Sigma-Aldrich, Schnelldorf Germany) was added to each tube, and the samples were centrifuged for 10min. The supernatants were collected and frozen at -80°C until assayed for IgA by ELISA (Bethyl laboratories Inc. Montgomery, Texas) within 45 months (when all samples were collected) as follows: a 96 well plate was coated overnight at 4°C with a 1:100 dilution of goat anti-canine IgA, affinity purified in 50μl of borate buffer (6.2g H_3_BO_3_/l, 9.54g Na_2_B_4_O_7_ 10H_2_O/l and 4.4g NaCl/l, pH7) and then washed with PBS-Tween-20. Free binding sites were blocked with 100μl of PBS containing 5% fetal calf serum and 0, 1% Tween-20 (ELISA buffer) for 1h at 37°C. Duplicate fecal extracts were incubated with ELISA buffer (final volume 50μl) for 2h at 37°C and then washed with PBS-Tween-20. The plate was incubated with a 1:10000 dilution of polyclonal goat anti-canine IgA conjugated with horseradish peroxidase in ELISA buffer (final volume 50μl) for 1h at 37°C, washed with PBS-Tween-20 and developed with 50μl of the TMB peroxidase substrate system according to the manufacturer´s instructions. The reaction was stopped with 50μl of 1M-phosphoric acid. Colour development was read at 450nm (BioTek Synerge H1 Hybrid Reader), and results expressed as μg/ml using a canine IgA standard. The concentration of fecal IgA was adjusted against total protein content in feces and expressed as μg IgA/μg total protein (TP).

### Sampling and measurement of antibodies in serum

Blood samples were collected from the cephalic vein. Before centrifugation samples were left to clot for at least 30 min at room temperature. After centrifugation (10 min, 7,200 rpm), serum was collected and frozen. All samples were frozen at -80°C within 48 hours. Between collection and freezing at -80°C, the samples were stored in dry ice or in -25°C freezer.

Serum was assayed for IgA, total IgE, and IgG against CDV within 45 months by ELISA (Bethyl laboratories Inc. Montgomery, Texas) as follows: a 96 well plate was coated overnight at 4°C with a 1:100 dilution of goat anti-canine IgA, IgE (Bethyl laboratories Inc., Montgomery, Texas) or CDV (VMRD, Inc.) Affinity purified in 50ul of borate buffer (6.2g H_3_BO_3_/l, 9.54g Na_2_B_4_O_7_ 10H_2_O/l and 4.4g NaCl/l, pH7) and then washed with PBS-Tween-20. Free binding sites were blocked with 100ul of PBS containing 5% fetal calf serum and 0.1% Tween-20 (ELISA buffer) for 1h at 37°C. Duplicate serum samples were incubated with ELISA buffer (final volume 50μl) for 2h at 37°C and then washed with PBS-Tween-20. The plate was incubated with a 1:10000 dilution of polyclonal goat anti-canine IgA conjugated with horseradish peroxidase in ELISA buffer (final volume 50μl) for 1h at 37°C, washed with PBS-Tween-20 and developed with 50μl of the TMB peroxidase substrate system according to the manufacturer´s instructions. The reaction was stopped with 50ul of 1M-phosphoric acid. Colour development was read at 450nm (BioTek Synerge H1 Hybrid Reader), and results expressed as μg/ml using a canine standard. The vaccine response was calculated as the difference in concentration of IgG against CDV between 7 weeks and 13 months. All results were expressed in this paper as g/L.

### Statistical data analysis

The relationships between the microbiota and metadata variables were explored using Orthogonal partial least square method with discriminant analysis (OPLS-DA) [18,19] using SIMCA-P+ and MATLAB routines. OPLS-DA is a constrained multivariate model meaning that the program is aware of the grouping and/or clustering of the samples and can answer if there are differences between treatments.

A standard 7-fold cross validation method was applied to establish the robustness of the models. In OPLS-DA models the Q^2^ are reported. The cross-validation parameter, Q^2^ (which can range from -1 to +1), represents the predictability of the models and is used to test the validity of the model against over-fitting. A Q^2^ value >0.6 indicates that differences between groups that are significantly different. Analysis of variance testing of cross-validated predictive residuals (CV-ANOVA) was also applied for each model and the ANOVA tables were reported. CV-ANOVA is another diagnostic tool for assessing the reliability of OPLS-DA models [20]. Pairwise OPLS-DA were applied to sample clusters (age, treatment, living area) with unit-variance scaling (each parameter has a mean of zero and a variance of one). Pairwise OPLS-DA models were generated with 1 predictive component, and 2 orthogonal components to discriminate between the groups. In order to detect any correlations between fecal IgA and microbiota, the fecal IgA data from puppies at all ages were divided into three percentiles, where the lower and upper 30% were regarded as low and high fecal IgA respectively.

To calculate the Shannon diversity index, the OTU table was first rarefied at the depth level of 500–550 sequences for 10 iterations per sample. Shannon index was calculated for each rarefied OTU table. Mann Whitney U test was performed to compare the diversity indexes between groups and the P values and mean +/- SD were reported. Mann Whitney U test was also performed to test for similarities in microbiota composition between mothers and puppies (based on weighted unifrac distances).

Linear discriminant analysis (LDA) effect size (LEfSe) was utilized to identify differentially abundant bacterial taxa. Linear discriminant analysis effect size algorithm is a high-dimensional class comparisons with a particular focus on metagenomic analyses [21]. LEfSe first determines the features (OTUs in this study) which are most likely to explain differences between classes by using nonparametric factorial Kruskal-Wallis sum-rank test. Then, LEfSe uses LDA to estimate the effect size of each differentially abundant feature. It can also provide a mapping of the differences to taxonomic or functional trees.

Similarities between littermates and unrelated dogs were tested using an analysis of similarity (ANOSIM) based on weighted UniFrac distance, where a p-value of <0.05 was regarded as a significant difference.

R (R Core Team, 2012) and *lme4* [22] were used to perform linear mixed effect analyses on the change of immunoglobulins over time and the effect of probiotic treatment upon immunoglobulins. Linear mixed effects models allowed us to control for this non-independence. For all the linear mixed models, litter membership was entered as a random effect where the intercept was allowed to vary between litters. The results were presented as the estimated population mean differences based on the model (β). P-values were obtained by Wald Z-tests.

For all analyses, data with a z-score less than -3 or greater than 3 was regarded as an outlier and was not included in the analyses. Level of significance was set to *p* = 0.05.

## Results

This study included 30 bitches and their 184 offspring, of which 168 completed the study. Fourteen dogs were excluded due to unrelated medical reasons and two dogs due to behaviour problems. Eleven of the 16 excluded puppies were excluded before 13 months of age. One litter (n = 2) in the La1-group and one litter (n = 2) in the placebo-group were delivered by caesarean section. It was too few dogs to do any separate analyses on this group. However, the four pups born by caesarean section were not outliers in the data.

### Gut microbiota in puppies and their mothers

Pyrosequencing of amplicons prepared from the V123 and V456 region of the 16 rRNA genes identified 306 unique operational taxonomic units (OTU) (S1 Table).

**Fig 1:**
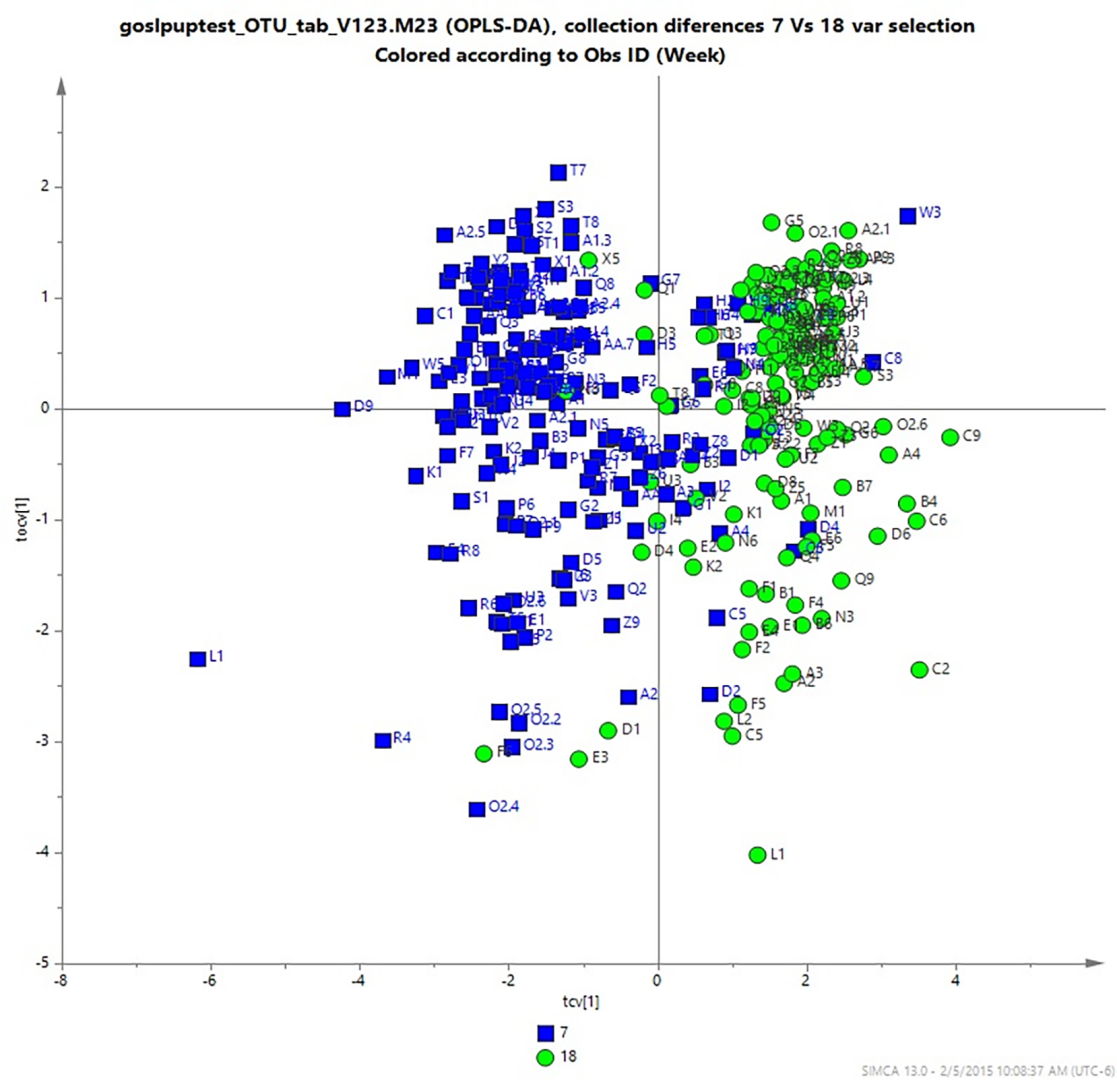
An OPLS-DA revealed differences in composition of the microbiota of dogs related to age. The OPLS-DA plot shows a comparison of the fecal microbiota in 7 weeks old puppies (blue) and when they becomes 15–18 months old (green), OPLS-DA; Q^2^ = 0.61.

The composition of the microbiota in puppies showed a clear age-related structure with a significant difference between 7 weeks old puppies and dogs at 15–18 months of age (OPLS-DA; Q^2^ = 0.61) (Fig 1).

**Fig 2:**
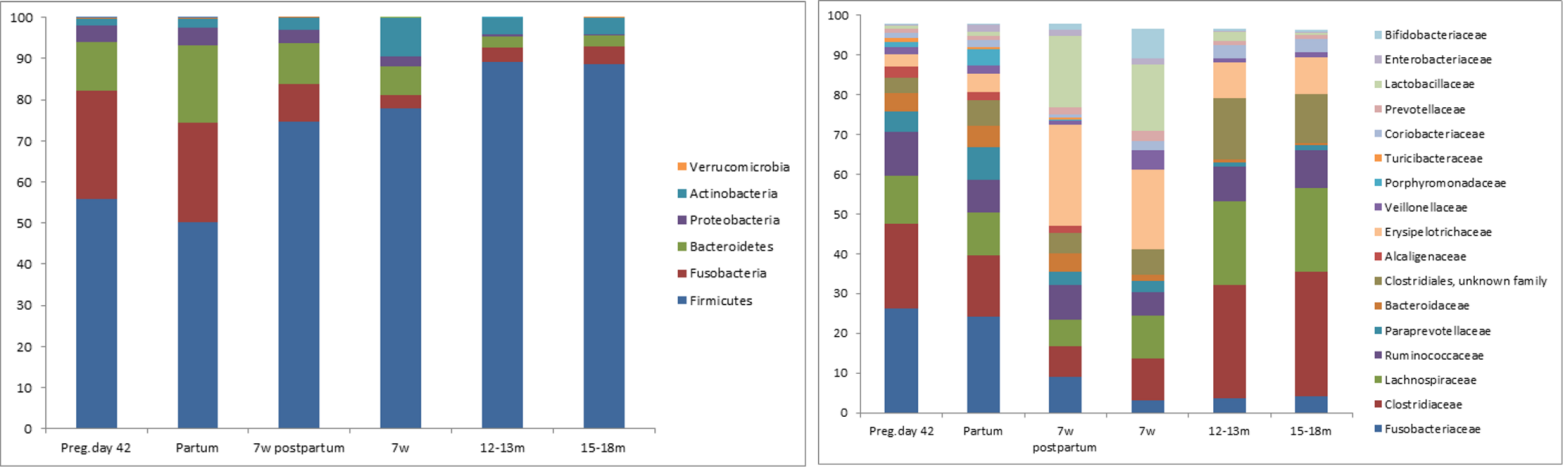
Relative abundance of bacteria phyla (a) and family (b) in feces from bitches at pregnancy day 42, partum and 7 weeks postpartum and from puppies at 7 weeks, 12–13 months and 15–18 months of age. Families with relative abundance >1% are included.

**Fig 3:**
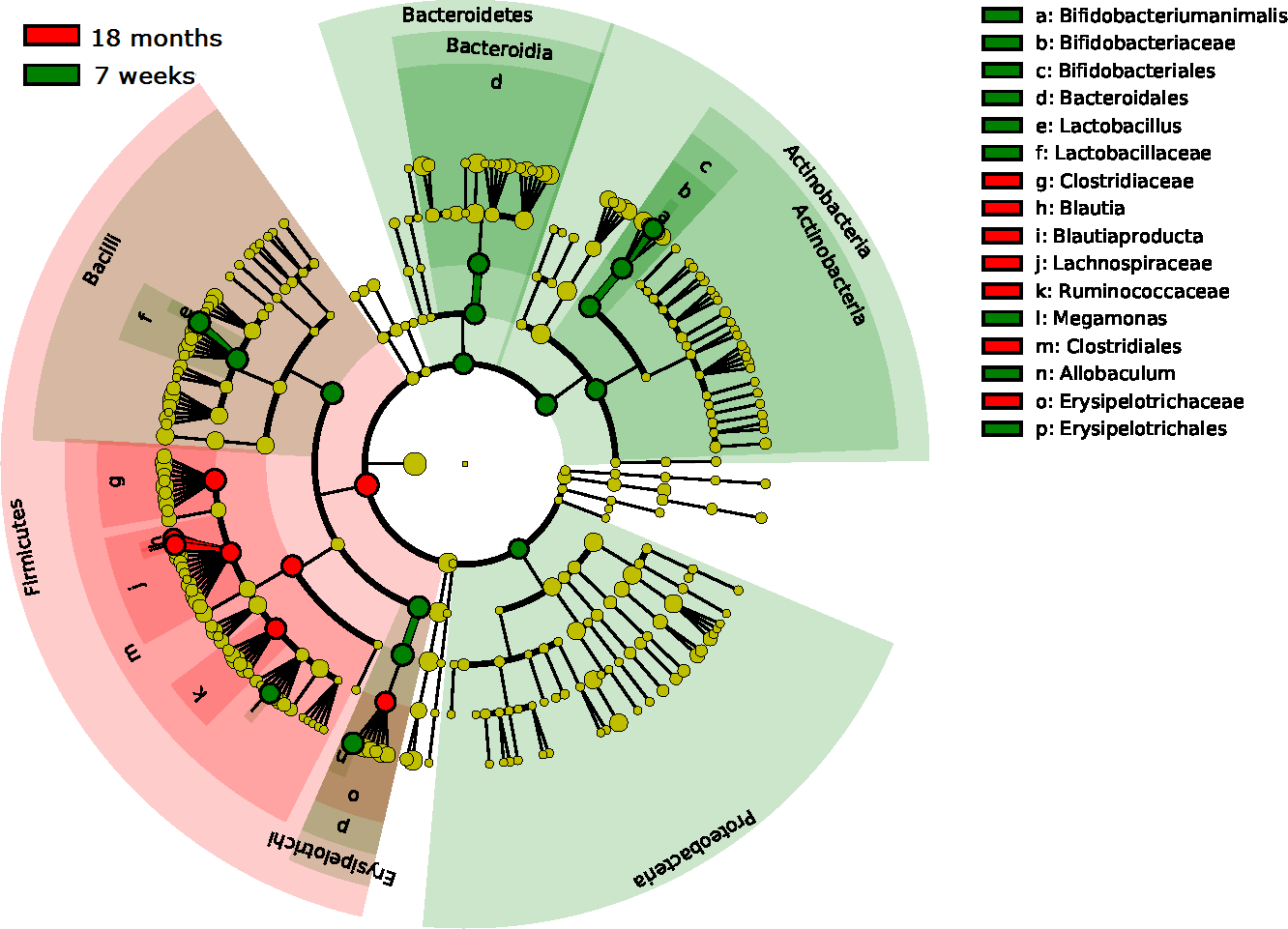
Linear discriminant analysis (LDA) effect size (LEfSe) showing differentially abundant bacterial taxa between 7 weeks and 15–18 months old dogs. Taxonomic groups significantly enriched in 7 weeks old puppies are indicated with green whereas the taxa enriched in 15–18 months are shown in red. P-value = 0.05 and absolute LDA score >4.0.

Firmicutes was the most dominant phylum at all ages with a relative abundance of 78–89% whereas Actinobacteria was the second most dominant phylum at all ages with a relative abundance of 4–9%. (Fig 2A). The abundance of three families, Clostridiaceae, Erysipelotrichaceae (unidentified genus) and Lachnospiraceae increased from puppyhood to adulthood (15–18 months of age) whereas Erysipelotrichaceae (genus Allobaculum), Lactobacillaceae and Bifidobacteriaceae decreased from puppyhood to adulthood (Fig 3). The dramatic change in composition of the microbiota from young to adult, was not reflected in diversity. The Shannon diversity index was similar from 7 weeks up to 15–18 months of age (3.28±0.63 to 3.02±0.81).

**Fig 4:**
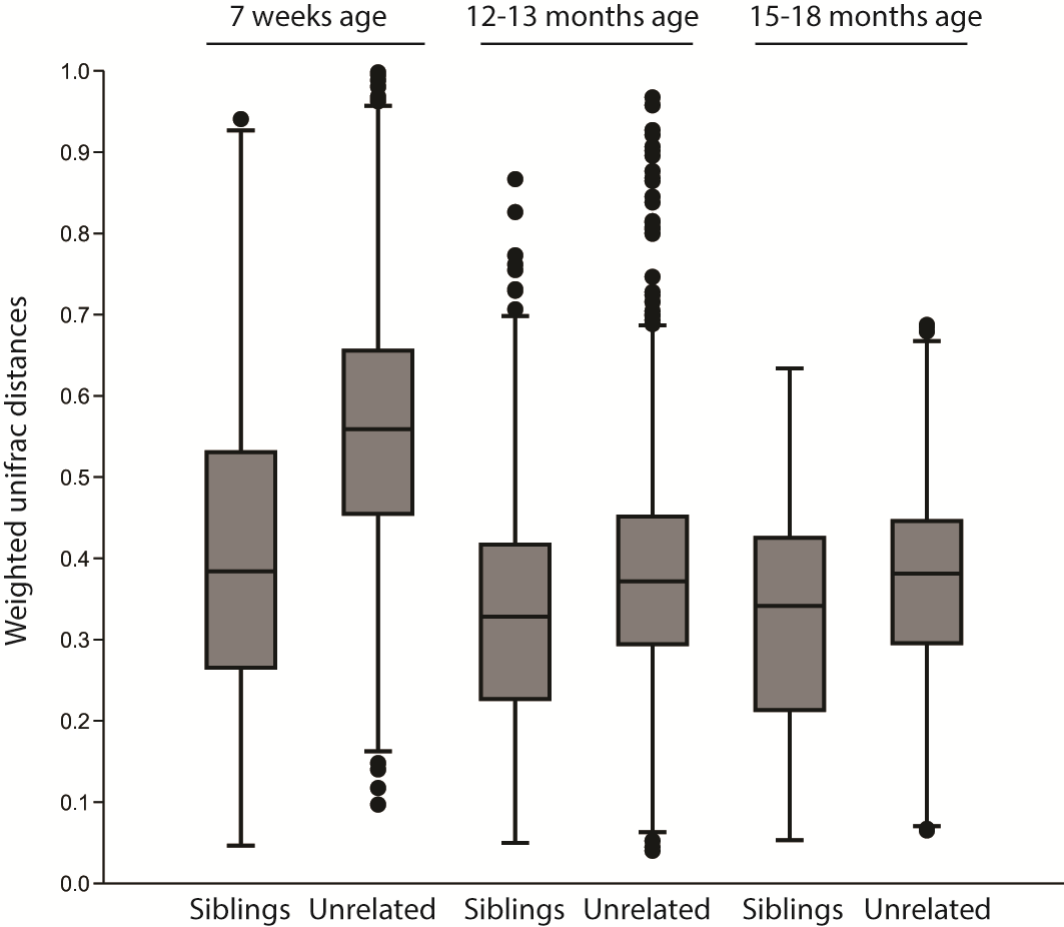
Boxplot showing the distribution of weighted unifrac distances comparing the microbiota between siblings and unrelated dogs at different ages. A distance of 0 represents an identical composition of the microbiota whereas 1 represents a total dissimilarity.

There was a strong litter effect at 7 weeks of age when the puppies lived in the same environment (ANOSIM analysis on weighted UniFrac distances: *R* = 0.49, *p* = 0.001). This litter effect was less obvious, but still significant, at 18 months of age when the dogs lived in different environments but still fed the same diet (weighted UniFrac *R* = 0.17, *p* = 0.001). The distribution of weighted Unifrac indexes between littermates and unrelated dogs for different ages are shown in Fig 4.

**Fig 5:**
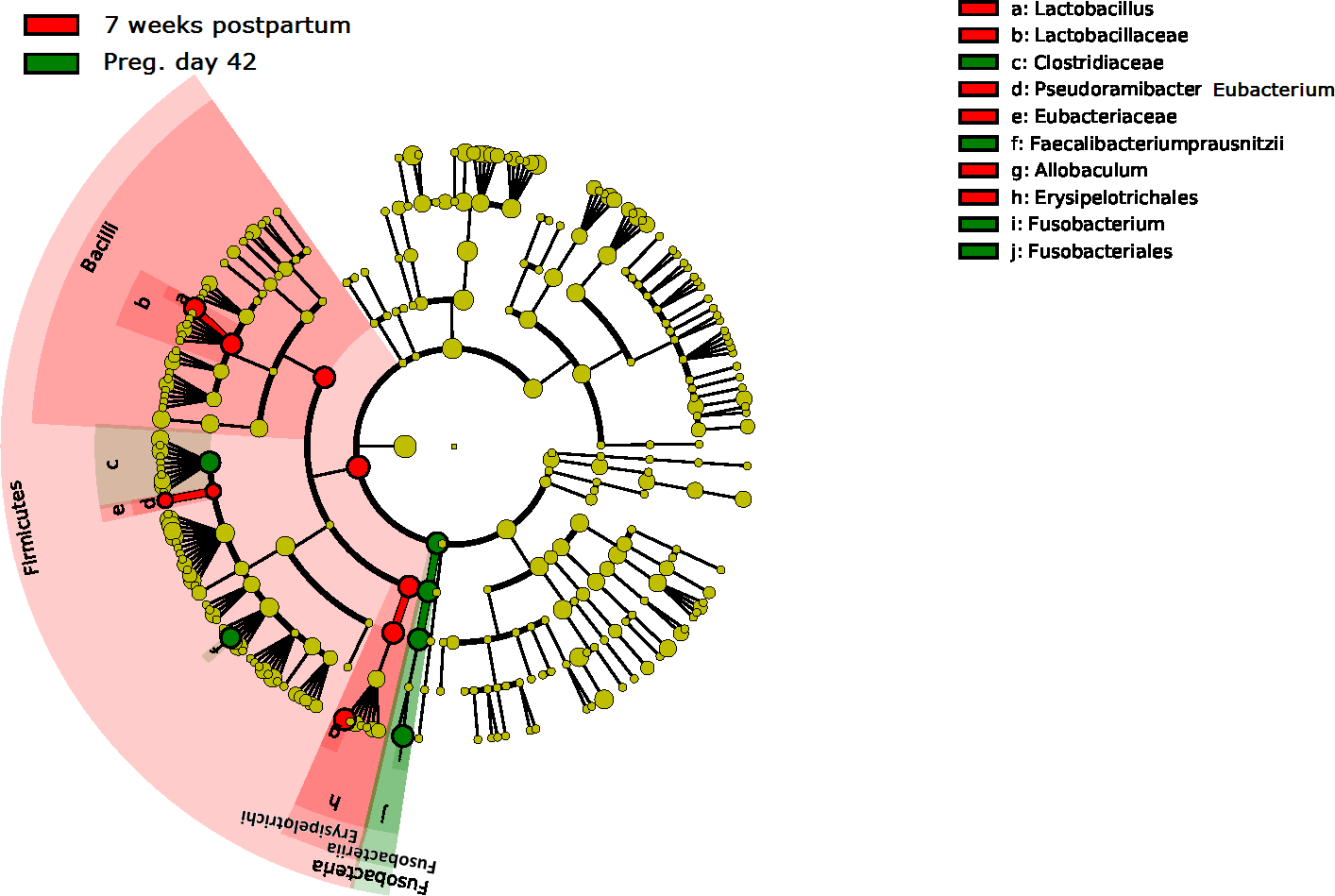
Linear discriminant analysis (LDA) effect size (LEfSe) showing differentially abundant bacterial taxa between pregnancy day 42 and 7 weeks postpartum. Taxonomic groups significantly enriched at pregnancy day 42 is shown in green and the taxa enriched 7 weeks postpartum are shown in red. P-value = 0.05 and abs LDA score >4.0.

**Fig 6:**
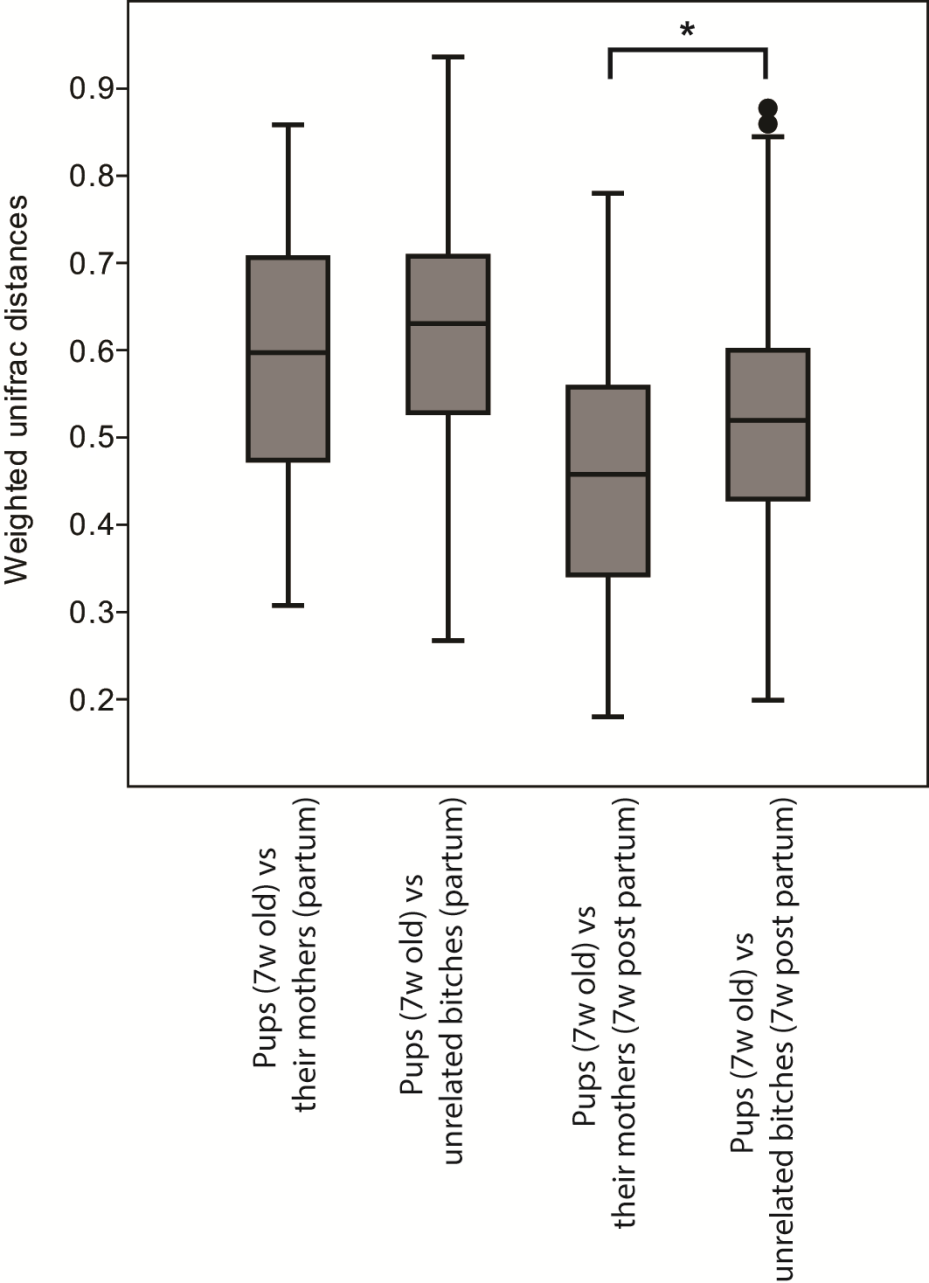
Boxplot showing the distribution of weighted unifrac distances comparing the composition of the microbiota in 7 weeks old puppies with their mothers at partum and 7 weeks postpartum, and with unrelated bitches at partum and 7 weeks postpartum. A distance of 0 represents an identical composition of the microbiota whereas 1 represents a total dissimilarity.

Firmicutes was the predominating phylum also in the bitches at all sampling points with relative abundances of 50–75% (Fig 2A). The bacterial community structure in bitches was stable from pregnancy day 42 to partum, but was shifted after whelping (between partum and 7 weeks postpartum; OPLS-DA, Q^2^ = 0.64 and pregnancy day 42 to 7 weeks postpartum; OPLS-DA, Q^2^ = 0.57). During this period, Erysipelotrichaceae and Lactobacillaceae were most increased, while Fusobacteriaceae and Clostridiaceae were most decreased (Fig 5). The microbial diversity increased from pregnancy day 42 to 7 weeks postpartum (Shannon´s diversity index: 3.76±0.41 to 4.03±0.37, p<0.01). The diversity was higher in the mothers than in the puppies at all ages. Interestingly, the composition of the microbiota also differed between the mothers 7 weeks postpartum and the 15–18 months old dogs (Q^2^ = 0.84) where the young dogs had more Clostridiaceae and Coriobacteriaceae, and the mothers had more Erysipelotrichaceae and Alcaligenaceae (Fig 2B). The composition of the fecal microbiota in bitches was more similar to the microbiota of puppies at 7 weeks postpartum than at partum. The 7 weeks old puppies were no more similar to their mothers than to unrelated bitches at partum. However, the puppies were significantly (P < 0.001) more similar to their mothers than to unrelated bitches at 7 weeks postpartum (Fig 6).

### The effect of environmental factors on fecal microbiota and immunoglobulins

The bitches were treated daily with *Lactobacillus johnsonii* NCC533 (La1) from pregnancy day 42 until the puppies were eight weeks old. Puppies were treated between 3–12 weeks of age. Supplementation of the probiotic strain (La1) did not influence the diversity or composition of the microbiota in either the bitches or the puppies. The probiotic treatment did not affect the levels of serum IgA, total serum IgE, fecal IgA in bitches or puppies or the vaccine response.

Diversity was significantly affected by living environment (countryside, small cities or big cities). Dogs living in big cities during their first 1.5 year of life had higher diversity compared to dogs living in small cities (3.36±0.63 vs 2.95±0.81, p<0.01) or at the countryside (3.36±0.63 vs 2.91±0.83, p<0.01). This difference was not seen at 7 weeks when all puppies lived at the kennel. The structural composition of the fecal microbiota was not significantly affected by living environment.

## Discussion

This is the first study to describe the fecal microbiota in a large number of dogs of the same breed, from the same kennel under well controlled natural conditions -giving us a unique opportunity to study the effects of age, relatedness, reproductive stage, living area and the effects of a characterized probiotic strain on the fecal microbiota.

We found that Firmicutes, Bacteroidetes, Fusobacteria and Actinobacteria were the predominant phyla in feces in puppies as well as in pregnant and lactating bitches. This is in accordance with earlier studies where a similar pattern has been described in adult dogs using pyrosequencing of fecal samples [23–25]. Although the predominant phyla are similar, proportions vary among these studies. Several sources can contribute to such variability, including breed, age, living conditions, diet and methodology. In our study, the microbial diversity was similar from 7 weeks to one year of age, although the composition of the fecal microbiota changed during this period with Erysipelotrichaceae being the most abundant family at 7 weeks of age and Clostridiaceae at one year of age. Litter mates had a more similar fecal microbiota profile compared to unrelated dogs, especially at 7 weeks of age. This could be explained by a more heterogenic environment at 13 and 18 months of age, compared to 7 weeks where the puppies lived in the same place. Our results are in accordance with the results of Hand et al. [4] who showed that 11 closely related miniature Schnauzer dogs had a more similar fecal microbiota profile compared to unrelated dogs within the same breed. By contrast, Middelbos et al. [23] could not find any correlation in a study where they compared 3 pairs of littermates. However, our study compared a much larger number of litters (n = 30) and this may account for the discrepancies between studies. The low sequencing depth in our study could also be a reason for different results between the studies. There were more pronounced differences in the composition of the fecal microbiota among younger than older dogs. This could be explained by a greater variation in diet between litters at 7 weeks of age, were some of the litters were weaned and only had solid food, while others were still suckling. The composition of microbes and oligosaccharides in the milk may also have had an effect.

In our study we observed a significant change in the relative abundance of different fecal bacteria during lactation (from partum to 7 weeks postpartum), but not during the last trimester of pregnancy. We also observed an increase of diversity (Shannon´s index) from pregnancy day 42 to 7 weeks postpartum. *Lactobacillus* was one of the genus that increased during lactation (in the probiotic as well as the placebo group). This genus was also higher in relative abundance in 7 week old puppies (probiotic and placebo groups) compared to young adults. Canine milk contains lactobacilli and may be a natural source of these potentially probiotic bacteria for the suckling puppy [26]. In humans [27,28] and mice [29] it was shown that during lactation, cells of the intestinal lymphoid tissue travelled to the mammary glands through the lymphatic system and peripheral blood, transferring maternal microbiota to the newborn via milk. This entero-mammary pathway could be a possible route in which maternal probiotic treatment during lactation affected the microbiota in suckling puppies. However, we could not detect a difference in the amount of lactobacilli in the fecal microbiota of puppies between the La1-group and the placebo group.

There was no difference in composition of the fecal microbiota between mothers and unrelated bitches at partum when comparing with 7 week old puppies. Our findings are similar to those of Koren et al. [30] in humans where they showed that children´s microbiotas (at all ages) were no more similar to their own mothers´ than unrelated mothers´. However, the puppies were significantly more similar to their mothers than to unrelated bitches at 7 weeks postpartum. This could be explained by the mothers´ behaviour of eating their puppies´ stool, making the maternal microbiota more similar to the microbiota of their puppies. That could also explain why the relative abundance of *Lactobacillus* spp. increased in the bitches during lactation.

As far as we know, this is the first and only study comparing the gut microbiota in pregnant and lactating bitches. Koren et al. [30] showed that the composition of the gut microbiota in women changes dramatically during pregnancy but are then stabilized during first month postpartum. This finding was supported by Carrothers et al. [31], Hesla et al. [32] and Jost et al. [33] who found that the microbiota in lactating women was relatively stable in the postpartum period. The results from the human studies are in contrast to our study where we found a significant change in the bitch fecal microbiota during lactation, which–once again–could be explained by the mothers´ behaviour of eating their puppies stool.

Our study is the first to show that the living environment affects the fecal microbiota of dogs. We observed differences between dogs growing up in the countryside compared to dogs living in cities. Dogs growing up in big cities had higher diversity than dogs living in small cities and at the countryside. These differences were not observed at 7 weeks of age when all puppies shared the same environment at the kennel, indicating that living area is the affecting factor in this aspect. Dicksved et al. [34] showed that anthroposophically raised children had higher diversity of their fecal microbiota compared to farm children, indicating that living conditions affect the diversity of human fecal microbiota. It was, however, not possible to pinpoint the responsible factors in their lifestyle that contributed to this difference. One important factor could be the diet, since the different lifestyles are related to consumption of different food. In our study, food was standardized throughout the whole study period which minimized the effect of diet. The impact of lifestyle upon the human gut microbiota has also been shown by Martinez et al. [35] who compared the fecal microbiota in adults from non-industrialized regions of Papua New Guinea with that of United States residents. They showed that Papua New Guineans had a fecal microbiota with higher diversity, lower inter-individual variation and different abundance profiles. However, dogs ingest more environmental microbes than humans because of their grooming habits, which might affect their fecal microbiota. Dogs living in big cities are often exposed to many different environments and a wide range of microbes, which might affect the microbial diversity. We also suspect that dogs living in big cities are more prone to travel around in different milieus, compared with dogs living at the countryside. Furthermore, the hygienic lifestyle of humans living in cities may not be reflected in their dogs.

Pre- and postnatal treatment with the probiotic La1 did not alter the composition of the fecal microbiota or diversity in either puppies or bitches. This is in accordance with results from the study of Garcia-Mazcorro et al. [36], who could not observe any changes in the fecal microbiota of healthy adult dogs (n = 12) after 3 weeks of treatment with a multi-species symbiotic (Proviable®-DC). Roos et al. [37] treated infants with *Lactobacillus reuteri* for three weeks and could not detect any significant changes in the composition of their fecal microbiota. However, the fecal microbiota may not be a representative marker to display how the microbiota in different parts of the gut is affected by the treatment. Samples from different parts of the gut would be needed to answer that question. Furthermore, a higher sampling depth would yield more extensive information regarding the composition and diversity of the microbiota. Besides the absence of impact on the intestinal microbiota, probiotic supplementation did not affect the levels of serum IgA and fecal IgA in our study, which is in accordance with the findings of Garcia-Mazcorro et al. [36]. Benyacoub et al. [10] fed laboratory dogs with *Enterococcus faecium* (SF68) daily from weaning up to one year of age. They showed that the treatment increased vaccine (CDV) response and amount of circulating IgA and fecal IgA, indicating an immune-stimulatory effect. However, we used another probiotic strain and a shorter treatment period which might account for our absence of a treatment effect.

Limitations of this study include the long interval time between first and second sample collection in puppies, the differences in diet and living conditions in bitches before the study, different length of the lactation period between litters, and a short treatment period in puppies. The treatment of puppies could have been more effective if started at birth, and in a larger study population with enough statistical power. That might have made it possible to detect the effect of probiotic treatment upon outcome of immune-related diseases. Biopsies from the small intestine would also give valuable information regarding the effects of probiotic treatment upon the gut microbiota. A methodological limitation is the low sequencing depth. Further studies should be focused on earlier treatment of puppies and with different probiotic strains, and in a larger study population with enough statistical power. That might have made it possible to detect the effect of probiotic treatment upon outcome of immune-related diseases.

In conclusion, we were able to describe the composition of gut microbiota in dogs and how it changes in different life stages including pregnancy, lactation and growth. Litter mates had a more similar fecal microbiota compared to unrelated dogs. We observed a change in the relative abundance of different bacteria during lactation, and an increase of diversity from pregnancy to end of lactation. We also found that the diversity of fecal microbiota was affected by living environment but we were unable to demonstrate an effect of pre and postnatal exposure to the chosen strain of probiotics.

Our findings provide a better understanding of the canine fecal microbiota in growing dogs as well as in pregnant and lactating bitches. Our results provide information to an area within canine microbiology which is not studied before -this is the first study to describe the gut microbiota in pregnant and lactating bitches and their offspring in a large well-defined study population. This extensive trial, with a large study population, born and raised under controlled conditions, provided us with a large amount of data, useful for further research on the relationship between the microbiome influences at an early age and immune function later in life.

## Supporting information

S1 Table OTU-table. (XLSX)

## References

[1] Suchodolski JS. 2011 Intestinal microbiome of dogs and cats: a bigger world than we thought. Vet. Clin. Small Anim. 41: 261–272. doi: 10.1016/j.cvsm.2010.12.006 21486635 10.1016/j.cvsm.2010.12.006 PMC7132526

[2] Benno Y, Nakao H, Uchida K, Mitsuoka T. 1992 Impact of the advances in age on the gastrointestinal microflora of beagle dogs. J. Vet. Med. Sci. 54: 703–706 1391181 10.1292/jvms.54.703

[3] Hooda S, Minamato Y, Suchodolski JS, Swanson KS. 2012 Current state of knowledge: the canine gastrointestinal microbiome. Anim Health Res Rev. 13: 78–88. doi: 10.1017/S1466252312000059 22647637 10.1017/S1466252312000059

[4] Hand D, Wallis C, Colyer A, Penn CW. 2013 Pyrosequencing the canine faecal microbiome: breadth and depth of biodiversity. PLoS One. 8 doi: 10.1371/journal.pone.0053115 23382835 10.1371/journal.pone.0053115 PMC3561364

[5] Strachan DP. 1989 Hay fever, hygiene and household size. BMJ. 299: 1259–1260 2513902 10.1136/bmj.299.6710.1259 PMC1838109

[6] Björkstén B. 2009 Disease outcomes as a consequence of environmental influences on the development of the immune system. Curr Opin Allergy Clin Immunol. 9:185–189 doi: 10.1097/ACI.0b013e32832abfc2 19398907 10.1097/ACI.0b013e32832abfc2

[7] Björkstén B, Sepp E, Julge K, Voor T, Mikelsaar M. 2001 Allergy development and the intestinal microflora during the first year of life. J Allergy Clin Immunol. 108: 516–520 doi: 10.1067/mai.2001.118130 11590374 10.1067/mai.2001.118130

[8] Prescott SL, Björkstén B. 2007 Probiotics for the prevention or treatment of allergic diseases. J Allergy Clin Immunol. 120: 255–262 doi: 10.1016/j.jaci.2007.04.027 17544096 10.1016/j.jaci.2007.04.027

[9] Fuller R. 1986 Probiotics. Society for Applied Bacteriology Symposium Series. 15: 1S–7S 3107133

[10] Benyacoub J, Czarnecki-Maulden GL, Cavadini C, Sauthier T, Anderson RE, Schiffrin EJ, et al 2003 Supplementation of food with enterococcus faecium (SF68) stimulates immune functions in young dogs. J Nutr. 133:1158–1162 12672936 10.1093/jn/133.4.1158

[11] Vilson Å, Bonnett B, Hansson-Hamlin H, Hedhammar Å. 2013 Disease patterns in 32,486 insured German shepherd dogs in Sweden: 1995–2006. Veterinary Record. 173 doi: 10.1136/vr.f961 23812178 10.1136/vr.101577

[12] Inoue R, Nishio A, Fukushima Y, Ushida K. 2007 Oral treatment with probiotic Lactobacillus johnsonii NCC533 (La1) for a specific part of the weaning period prevents the development of atopic dermatitis induced after maturation in model mice, NC/Nga, Br. J. Dermatol. 156: 499–509 doi: 10.1111/j.1365-2133.2006.07695.x 17300240 10.1111/j.1365-2133.2006.07695.x

[13] Kaburagi T, Yamano T, Fukushima Y, Yoshino H, Mito N, Sato K. 2007 Effect of Lactobacillus johnsonii La1 on immune function and serum albumin in aged and malnourished aged mice. Nutrition. 23: 342–350 doi: 10.1016/j.nut.2007.02.001 17367996 10.1016/j.nut.2007.02.001

[14] Sanchez M, Darimont C, Drapeau V, Emady-Azar S, Lepage M, Rezzonico E, et al 2013 Effect of *Lactobacillus rhamnosus* CGMCC1.3724 supplementation on weight loss and maintenance in obese men and women. Br J Nutr. 111: 1507–1519 doi: 10.1017/S0007114513003875 24299712 10.1017/S0007114513003875

[15] Caporaso JG, Kuczynski J, Stombaugh J, Bittinger K, Bushman FD, Costello EK, et al 2010 QIIME allows analysis of high-throughput community sequencing data. Nat Methods. 7: 335–336. doi: 10.1038/nmeth.f.303 20383131 10.1038/nmeth.f.303 PMC3156573

[16] Reeder J, Knight R. 2010 Rapidly denoising pyrosequencing amplicon reads by exploiting rank-abundance distributions. Nat Methods. 7: 668–669. doi: 10.1038/nmeth0910-668b 20805793 10.1038/nmeth0910-668b PMC2945879

[17] Edgar RC, Haas BJ, Clemente JC, Quince C, Knight R. 2010 UCHIME improves sensitivity and speed of chimera detection. Bioinformatics. 27: 2194–2200 10.1093/bioinformatics/btr381 PMC3150044 21700674

[18] Trygg J, Wold S. 2002 Orthogonal projections to latent structures (O-PLS). J Chemom. 16:119–128. doi: 10.1002/cem.695

[19] Ramadan Z, Xu H, Laflamme D, Czarnecki-Maulden G, Li QJ, Labuda J, et al 2014 Fecal Microbiome of Cats with Naturally Occurring Chronic Diarrhea Assessed Using 16S rRNA Gene 454-Pyrosequencing before and after Dietary Treatment. J Vet Intern Med. 28: 59–65 doi: 10.1111/jvim.12261 24592406 10.1111/jvim.12261 PMC4895530

[20] Eriksson L, Trygg J, Wold S. 2008 CV-ANOVA for significance testing of PLS and OPLS models, Journal of Chemometrics, Special Issue: Proceedings of the 10th Scandinavian Symposium on Chemometrics, SSC10, 22; 594–600

[21] Segata N, Izard J, Waldron L, Gevers D, Miropolsky L, Garrett WS, et al 2011 Metagenomic biomarker discovery and explanation. Genome Biology. 12: R60 doi: 10.1186/gb-2011-12-6-r60 21702898 10.1186/gb-2011-12-6-r60 PMC3218848

[22] Bates D.M, Maechler M, Bolker B. (2012) lme4: Linear mixed-effects models using S4 classes. R package version 0.999999–0.

[23] Middelbos IS, Vester Boler BM, Qu A, White BA, Swanson KS, Fahey GC Jr. 2010 Phylogenetic characterization of fecal microbial communities of dogs fed diets with or without supplemental dietary fiber using 454 pyrosequencing. PLoS ONE. 5 doi: 10.1371/journal.pone.0009768 20339542 10.1371/journal.pone.0009768 PMC2842427

[24] Handl S, Dowd SE, Garcia-Mazcorro JF, Steiner JM, Suchodolski JS. 2011 Massive parallel 16S rRNA gene pyrosequencing reveals highly diverse fecal bacterial and fungal communities in healthy dogs and cats. FEMS Microbiol Ecol. 76: 301–310. doi: 10.1111/j.1574-6941.2011.01058.x 21261668 10.1111/j.1574-6941.2011.01058.x

[25] Garcia-Mazcorro JF, Dowd SE, Poulsen J, Steiner JM, Suchodolski JS. 2012 Abundance and short-term temporal variability of fecal microbiome in healthy dogs. Microbiology Open. 1: 340–347. doi: 10.1002/mbo3.36 23170232 10.1002/mbo3.36 PMC3496977

[26] Martin E, Olivares M, Pérez M, Xaus J, Torre C, Fernández L, et al 2010 Identification and evaluation of the probiotic potential of lactobacilli isolated from canine milk. The Veterinary Journal. 185: 193–198 doi: 10.1016/j.tvjl.2009.04.014 19451012 10.1016/j.tvjl.2009.04.014

[27] Donnet-Hughes A, Perez P, Doré J, Leclerc M, Levenez F, Benyacoub J, et al 2010 3^rd^ International Immunonutrition Workshop Session 7: Prebiotics and probiotics usefulness against pathologies Potential role of the intestinal microbiota of the mother in neonatal immune education. Proceedings of the Nutrition Society. 69: 407–415 doi: 10.1017/S0029665110001898 20633308 10.1017/S0029665110001898

[28] Jost T, Lacroix C, Braegger C, Chassard C. 2014 Vertical mother-neonate transfer of maternal gut bacteria via breastfeeding. Environ Microbiol. 16: 2891–2904 doi: 10.1111/1462-2920.12238 24033881 10.1111/1462-2920.12238

[29] Perez PF, Dore J, Leclerc M, Levenez F, Benyacoub J, Serrant P, et al 2007 Bacterial imprinting of the neonatal immune system: lessons from maternal cells? Pediatrics 119: e724–e732. doi: 10.1542/peds.2006-1649 17332189 10.1542/peds.2006-1649

[30] Koren O, Goodrich JK, Cullender TC, Spor A, Laitinen K, Bäckhed HK, et al 2012 Host remodeling of the gut microbiome and metabolic changes during pregnancy. Cell. 150: 470–480. doi: 10.1016/j.cell.2012.07.008 22863002 10.1016/j.cell.2012.07.008 PMC3505857

[31] Carrothers JM, York MA, Brooker SL, Lackey KA, Williams JE, Shafii B, et al 2015 Fecal microbial community structure is stable over time and related to variation in macronutrient and micronutrient intakes in lactating women. J Nutr. 145: 2379–2388. doi: 10.3945/jn.115.211110 26311809 10.3945/jn.115.211110 PMC4580954

[32] Hesla HM, Stenius F, Jäderlund L, Nelson R, Engstrand L, Alm J, et al 2014 Impact of lifestyle on the gut microbiota of healthy infants and their mothers–the ALADDIN birth cohort. FEMS Microbiol Ecol. 90: 791–801 doi: 10.1111/1574-6941.12434 25290507 10.1111/1574-6941.12434

[33] Jost T, Lacroix C, Braegger C, Chassard C. 2014 Stability of the maternal gut microbiota during late pregnancy and early lactation. Curr Microbiol. 68: 419–27 doi: 10.1007/s00284-013-0491-6 24258611 10.1007/s00284-013-0491-6

[34] Dicksved J, Flöistrup H, Bergström A, Rosenquist M, Pershagen G, Scheynius A, et al 2007 Molecular fingerprinting of the fecal microbiome of children raised according to different lifestyles. Appl. Enivorn. Microbiol. 73: 2284–2289 10.1128/AEM.02223-06 PMC1855685 17293501

[35] Martinez I, Stegen JC, Maldonado-Gómez MX, Eren AM, Siba PM, Greenhill AR, et al 2015 The gut microbiota of rural papua new guineans: composition, diversity patterns, and ecological processes. Cell Rep. 11: 527–538 doi: 10.1016/j.celrep.2015.03.049 25892234 10.1016/j.celrep.2015.03.049

[36] Garcia-Mazcorro JF, Lanerie DJ, Dowd SE, Paddock CG, Grutzner N, Steiner JM, et al 2011 Effect of a mulit-species symbiotic formulation on fecal bacterial microbiome of healthy cats and dogs as evaluated by pyrosequencing. FEMS Microbio Ecol. 78: 542–554. doi: 10.1111/j.1574-6941.2011.01185.x 22067056 10.1111/j.1574-6941.2011.01185.x

[37] Roos S, Dicksved J, Tarasco V, Locatelli E, Ricceri F, Grandin U, et al 2013 454 Pyrosequencing analysis on faecal samples from a randomized DBPC trial of colicky infants treated with Lactobacillus reuteri DSM 17938. PLoS One. 8 doi: 10.1371/journal.pone.0056710 23468874 10.1371/journal.pone.0056710 PMC3585302

